# Supplementary material for: Conditional chemoconnectomics (cCCTomics) as a strategy for efficient and conditional targeting of chemical transmission
Source: eLife. 2024 Apr 30;12:RP91927. doi: 10.7554/eLife.91927 (PMC11060718; doi:10.7554/eLife.91927)
Supplement: Figure 3—source data 3. [file elife-91927-fig3-data3.zip › Figure 3 source data3 part 1.zip!/Figure 3-Source data 3-part1/1063-13-CRISPResso_Report_8cz_fsdr.zip!/CRISPResso_on_1063_14/2a.Nucleotide_percentage_quilt.pdf]

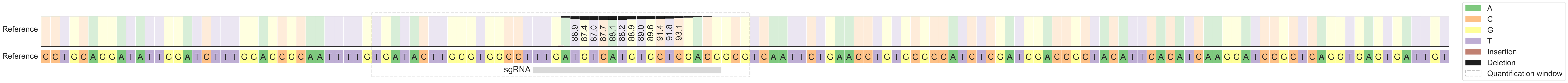
Reference
88.9
87.4
87.0
87.7
88.1
88.2
88.9
89.0
89.6
91.4
91.8
93.1
Reference
CCTGCAGGATATTGGATCTTTGGAGCGCAATTTTGTGATACTTGGGTGGCCTTTGATGTCATGTGCTCGACGGCGTCAATTCTGAACCTGTGCGCCATCTCGATGGACCGCTACATTCACATCAAGGATCCGCTCAGGTGAGTGATTGT
sgRNA
A
C
G
T
Insertion
Deletion
Quantification window
